# Challenges in diagnosing dementia in patients with a migrant background - a cross-sectional study among German general practitioners

**DOI:** 10.1186/s12875-019-0920-0

**Published:** 2019-02-25

**Authors:** Judith Tillmann, Johannes Just, Rieke Schnakenberg, Klaus Weckbecker, Birgitta Weltermann, Eva Münster

**Affiliations:** 1 Institute of General Practice and Family Medicine, Medical Faculty of the University of Bonn, Bonn, Germany; 2 Department of Health Services Research, Carl von Ossietzky University Oldenburg, Oldenburg, Germany

**Keywords:** General practitioner, Dementia, Diagnostics, Migration, GP, Public health

## Abstract

**Background:**

Diagnosing dementia, a syndrome affecting 35.6 million people worldwide, can be challenging, especially in patients with a migrant background. Language barriers and language-based diagnostic tools, cultural differences in the perception of the syndrome as well as restricted access to healthcare can influence medical care. For the first time in Germany, this study investigates whether German general practitioners (GPs) feel prepared to meet the diagnostic needs of these patient groups and whether there are challenges and support needs.

**Methods:**

A cross-sectional study among a random sample of 982 general practitioners in Germany was conducted from October 2017 to January 2018 (response rate: 34.5%). A self-developed, written, standardised questionnaire was used. Descriptive statistics as well as multiple logistic regression analyses were performed using data of 326 GPs.

**Results:**

Ninety-six percent of GPs reported having experienced barriers at least once. Uncertainties in diagnosing dementia in patients with a migrant background were indicated by 70.9%. There was no significant association between uncertainties in diagnosing dementia and GPs’ sociodemographic characteristics. The most frequently reported barriers were language barriers that affected or prevented diagnostics (89.3%) and information deficits in patients with a migrant background (59.2%). Shameful interaction or lack of acceptance of the syndrome was also common (55.5%). A demand for more information about the topic was expressed by 70.6% of GPs.

**Conclusions:**

Public health measures supporting GPs in their interaction with patients with a migrant background as well as information and services for dementia patients are needed. Efforts to facilitate access to interpreting services and to focus on people with a migrant background in healthcare are necessary.

**Trial registration:**

German Clinical Trials Register: DRKS00012503, date of registration: 05/09/2017 (German Institute of Medical Documentation and Information. German Clinical Trials Register (DRKS) 2017). Clinical register of the study coordination office of the University hospital of Bonn: ID530, date of registration: 05/09/2017 (Universitätsklinikum Bonn. Studienzentrum. UKB-Studienregister 2017).

## Background

### Scientific background and relevance

Diagnosing dementia in individuals with a migrant background can be associated with various difficulties from the perspective of the diagnosing person: people with a migrant background often do not speak the national language fluently or forget the second language. Hence, difficulties in applying language-based diagnostic instruments may arise [1–4]. Cultural facors may influence attitudes and coping with dementia. Dementia and mental diseases that are often tabooed, induce feelings of shame and are therefore difficult to address. Diagnosis, therapy options and help from outside the family is sometimes not accepted [5–9]. In some cultures, family plays a central role in caring for sick family members [8–10]. According to international and European studies, migrant background was associated with reduced access to health care [3, 8, 9, 11]. Associations between low health literacy and socio-economic standing and migrant status have already been shown [12].

The increasing life expectancy associated with the ongoing demographic change is causing a steep increase in the number of people with age-related diseases like dementia. An increase of currently 35.6 million patients with dementia worldwide (2010) to 42 million patients by 2040 is predicted [13, 14]. In Germany, a country with 82.4 million inhabitants, it is likely that the number of cases will increase from 1.55 million to 3 million in 2050 [15, 16]. At the same time, the number of people with a migrant background is growing rapidly in Germany (currently 18.56 million, 22.5%). This population group is also ageing steadily and is increasingly affected by dementia as well [16]. The German Federal Statistical Office (Destatis) defines a migrant background as follows: Either the person in question or at least one parent is born without German nationality [17]. Within this group, Turkey (15.1%), Poland (10.1%) and Russia (6.6%) are the most common countries of origin in Germany [16]. However, there is no official, uniform definition at global level. Destatis reports that there are 1.86 million people with a migrant background who are aged 65 years or older and are therefore at risk of developing dementia [16]. More specifically, the “Dementia Service Centre for People With Immigration History” reports that there are 108,000 individuals with a migrant background suffering from dementia in Germany (2015) [18].

GPs play a key role in diagnostics in Germany and are in the best position to raise awareness for all types of dementia. They examine a large number of patients and are generally the first point of contact for people with any health complaints. Therefore, they are able to identify treatable causes of the syndrome at an early stage to prevent irreversible health impairment.

According to European studies conducted by Nielsen et al., two thirds of physicians describe the diagnostics and classification of dementia in ethnic minorities as problematic [1]. On an international level, there is also evidence suggesting that dementia is underdiagnosed in immigrants and minority ethnic groups [3, 6, 19]. Despite the described increase in affected people worldwide and identified barriers in international studies, it is unknown whether doctors in Germany and other European countries are prepared to meet the diagnostic needs of these patient groups [1, 20]. Hence, the study “Barriers in GPs’ dementia diagnostics in patients with migration background” (BaDeMi) is the first of its kind in Germany to focus on identifying challenges in diagnosing dementia in people with a migrant background.

### Objectives

The aim of the study was to examine challenges in diagnosing dementia in patients with a migrant background in German GP practices. And if so, what challenges exist and are there information needs? Are sociodemographic characteristics of GPs associated with their confidence in diagnosing dementia in patients with a migrant background?

## Methods

### Study design

A cross-sectional study in a simple random sample without replacement of 982 GPs (response rate 34.5%; 339 GPs) was conducted. The exploratory study took place in general practitioners’ practices in North Rhine-Westphalia, the most densely populated state in western Germany (17.87 million inhabitants), from October 2017 to January 2018. North Rhine-Westphalia is by far the federal state with the highest number of people with a migrant background (5,036,000; 28.4%) [21]. The definition of a migration background of the German Federal Statistical Office was used: Either the person or at least one parent is born without the German nationality [17]. The standardised, self-administered, written survey included questions about GPs’ experience in diagnosing dementia in patients with a migrant background and ways to improve diagnostics and support physicians. Sociodemographic data of GPs, including age, sex, language skills and migration background was collected. Five-point Likert-type scales with responses ranging from ‘strongly disagree’ to ‘strongly agree’ as well as multiple-choice questions were used as response categories. The questions were developed based on a systematic literature search in medical databases and Google Scholar. The most common problems in the diagnostic process and in dealing with patients with a migrant background described in the international literature were included in the questionnaire as questions or answer categories. In addition, free text fields were added to describe further aspects. Few questions were based on a survey developed by Australian researchers of Wicking Dementia Research & Education Centre (University of Tasmania) within the scope of a collaboration [22]. The questions were translated using the method of back-translation by an English native speaker to ensure comparability. Before conducting the study, the questionnaire was pretested by general practitioners to identify possible sources of error. Validity and reliability were not further investigated. More detailed information on the process of questionnaire development are provided in the methods paper [23]. Address data of the physicians were provided by the ‘Association of Statutory Health Insurance Physicians North-Rhine’ upon request. The target population was contacted by the institute of general practice using a postal mail with the questionnaires enclosed. Two written, postal reminder procedures were carried out, each with a waiting period of 4 weeks. Informed consent to participate in the study was documented by answering and returning the pseudonymised questionnaire. The study has been registered at the German Clinical Trials Register (DRKS) (no. DRKS00012503) [24] and the clinical register of the study coordination office of the University hospital of Bonn (ID530) [25].

The following questions of the questionnaire were included in the analysis for the present study (translated from the German questionnaire). 5-point Likert scales ranging from “I don’t agree at all” to “I fully agree” ^(^^a^^)^ or “never” to “very frequently” ^(^^b^^)^ as well as multiple responses with additional free-text fields ^(^^c^^)^, multiple-choice-fields ^(^^d^^)^ and free-text fields ^(^^e^^)^ were used as response catagories:

- I feel confident in diagnosing dementia. ^a^
- I feel confident in diagnosing dementia in people with a migrant background. ^a^
- I feel confident about communicating the dementia diagnosis to a patient. ^a^
- I feel confident about communicating the dementia diagnosis to a patient with a migrant background. ^a^
- I have enough knowledge about local help centers that support dementia patients and their families. ^a^
- I have enough knowledge about local help centers that support dementia patients with a migrant background and their families. ^a^
- I have not been able to use cognitive short tests at least once due to these language difficulties between the patient with a migrant background and myself. ^a^
- What barriers have you ever experienced during dementia diagnostics? ^c^
- How have you so far dealt with language problems in dementia diagnostics between you and your patients with a migrant background? ^c^
- How often did these barriers and language problems prevent you from optimally treating a patient with a migrant background for dementia? ^b^
- Would you like to get more information on how to deal better with dementia patients with a migrant background? ^d^
- Which information are you personally interested in? ^c^
- Sociodemographic and practice-related paramaters: How old are you? ^e^, Which gender do you have? ^d^, Is your mother or father or were you born abroad? ^d^, How long have you been working as a general practitioner so far? ^e^, Please estimate: how high is the percentage of people with a migrant background among your patients? ^e^

### Participants

The target group of this study were general practitioners in North Rhine-Westphalia who were actively practicing medicine when the study was conducted. In order to meet the inclusion criteria, GPs had to be registered in the ‘Association of Statutory Health Insurance Physicians North-Rhine’ as a general practitioner on July 28th, 2017. In Germany, physicians have to be members of this association to be allowed to treat patients with statutory health insurance (87.7% of the population) [26].

### Statistical methods

The questionnaires were scanned using the data capture system TeleForm [27]. The software IBM SPSS Statistics (Version 22) was used for data analyses [28]. Descriptive statistics including frequencies with 95% confidence intervals, medians, means and standard deviations were calculated to evaluate GPs’ data. Multiple logistic regression analysis was conducted to examine the association between sociodemographic characterisitcs and GPs’ confidence in diagnosing dementia in patients with a migrant background. The dependent variable was dichotomised into the categories “I fully/rather agree/neutral” and “I fully/rather disagree”. The sociodemographic variables shown in Table 1 were used as independent variables to analyse whether characteristics of GPs and their practices are associated with problems in diagnosing dementia. Variables were included in the analysis simultaneaously. All independent variables were dichotomised to reduce the degrees of freedom (Table 2). Missing data in the dependent variable were excluded from analysis. Missings in independent variables were allocated to the reference category (largeste group) because they did not exceed a predetermined limit of 6%. Crude odds ratios (OR) with 95% confidence intervals (CI) were calculated. To control for confounding, odds ratios adjusted for age, gender, migration background and percentage of patients with a migrant background (aOR) with 95% CI were computed for all participants. Crude and adjusted odds ratios stratified by gender were computed to consider potential effect modification. A *p*-value < 0.05 was considered significant.

**Table 1:** Characteristics of the study population and prevalence of not feeling confident in dementia diagnostics in patients with a migrant background n= 326).

	Total study population		Prevalence of not feeling confident	
	n	(%) ^a,b^	n	(%; 95% CI) ^a,c^
Total	326	(100)	231	(70.9; 65.9–75.6)
Gender				
Female	153	(46.9)	109	(71.2; 64.0–78.5)
Male	173	(53.1)	122	(70.5; 63.7–77.4)
Age				
< 50	105	(32.2)	69	(65.7; 56.5–74.9)
> =50	221	(67.8)	162	(73.3; 67.4–79.2)
GP has a migrant background				
No	278	(85.3)	200	(71.9; 66.6–77.3)
Yes	48	(14.7)	31	(64.6; 50.6–78.6)
Estimated percentage of patients with a migrant background in the practice				
1–20%	251	(77.0)	174	(69.3; 63.6–75.1)
> 20%	75	(23.0)	57	(76.0; 66.1–85.9)

^a^Missing cases were allocated to the reference category of logistic regression (age: *n* = 9 (2.8%), gender: *n* = 0, GP has a migrant background: *n* = 4 (1.2%), estimated percentage of patients with a migrant background: *n* = 10 (3.1%)). Missings in the dependent variables were excluded (*n* = 11; 3.2%).

^b^column percentages; ^c^ row percentages.

**Table 2:** GPs lack of confidence in diagnosing dementia in patients with a migrant background

	OR (95% CI) total ^a^ (*n* = 326)	aOR (95% CI) total ^b^ (*n* = 326)	OR (95% CI) men ^a^ (*n* = 173)	aOR (95% CI) men ^b^ (*n* = 173)	OR (95% CI) women ^a^ (*n* = 153)	aOR (95% CI) women ^b^ (*n* = 153)
Gender						
Female	1.04 (0.64–1.67)	1.06 (0.66–1.72)	–	–	–	–
Male	*ref.*	*ref.*	–	–	–	–
Age						
< 50	0.70 (0.42–1.15)	0.68 (0.40–1.13)	0.60 (0.30–1.20)	0.59 (0.29–1.21)	0.82 (0.40–1.69)	0.78 (0.38–1.64)
> =50	*ref.*	*ref.*	*ref.*	*ref.*	*ref.*	*ref.*
GP has a migrant background						
No	*ref.*	*ref.*	*ref.*	*ref.*	*ref.*	*ref.*
Yes	0.71 (0.37–1.36)	0.69 (0.36–1.33)	0.47 (0.20–1.12)	0.46 (0.19–1.13)	1.17 (0.43–3.20)	1.21 (0.44–3.34)
Estimated percentage of patients with a migrant background in the practice						
1–20%	*ref.*	*ref.*	*ref.*	*ref.*	*ref.*	*ref.*
> 20%	1.40 (0.77–2.54)	1.50 (0.82–2.74)	0.92 (0.43–2.01)	1.12 (0.50–2.52)	2.41 (0.92–6.27)	2.45 (0.94–6.40)

^a^crude odds ratios with 95% confidence intervals (CI) estimated from logistic regression. Missing cases in the independent variables were allocated to the reference category (age: *n* = 9 (2.8%), gender: *n* = 0, GP has a migrant background: *n* = 4 (1.2%), estimated percentage of patients with a migrant background: *n* = 10 (3.1%)). Missings in the dependent variables were excluded (*n* = 11; 3.2%).

^b^Adjusted odds ratios with 95% confidence intervals (CI) estimated from logistic regression (adjustment for the other sociodemographic and practice-related determinants of the model, method: enter).

## Results

### Characteristics of the study population

**Fig. 1:**
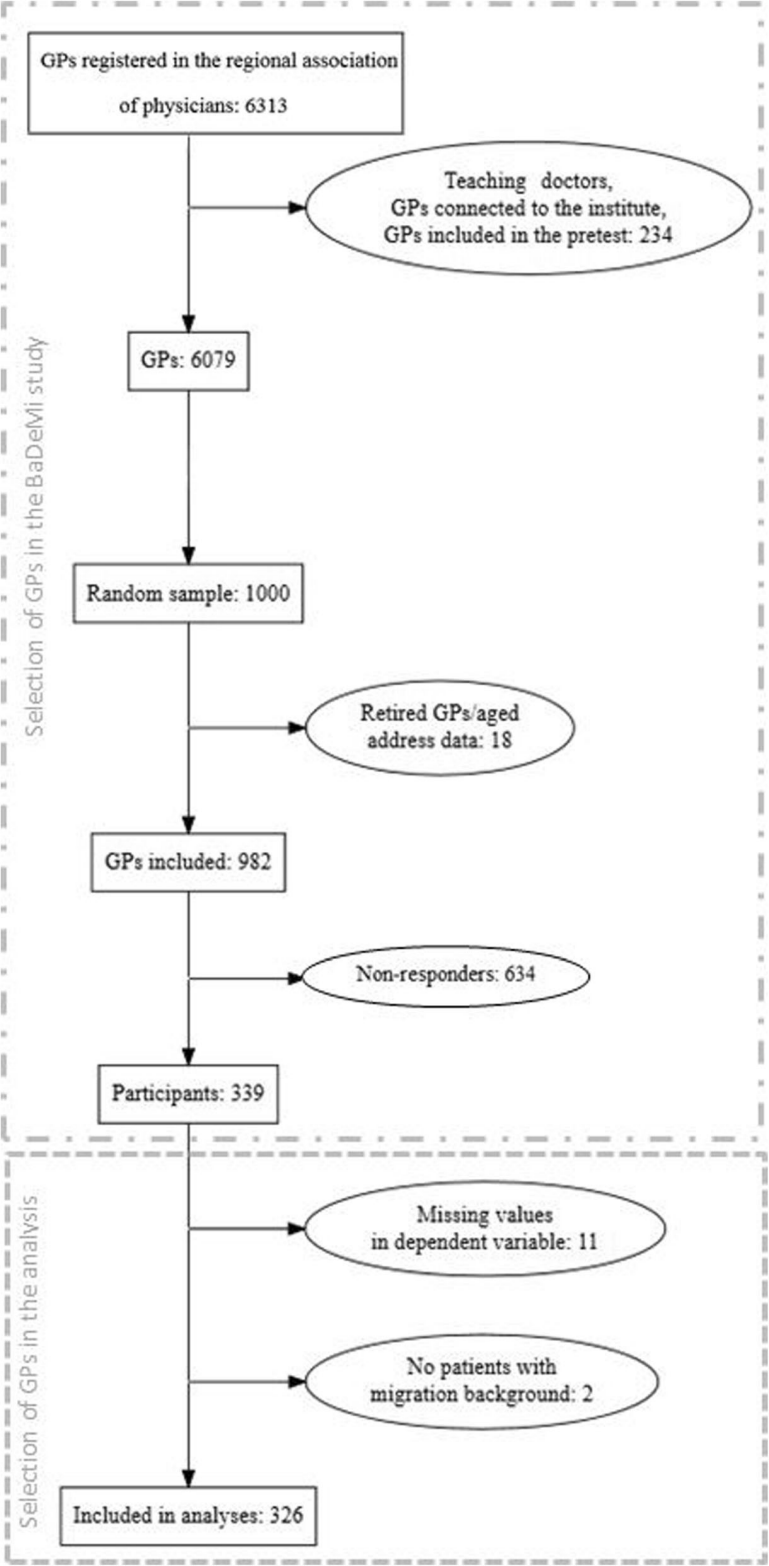
Flow diagram of study population: participating general practitioners

A response rate of 34.5% was achieved. Thirty percent of male and 36.2% of female GPs participated in the survey. A total of 326 GPs were included in the analyses as shown in Fig. 1. The mean age of participants was 53.5 years (SD = ±8.9). The average duration of practicing as a GP was 16.9 years (SD = ±10.0). The sex ratio of participants was nearly balanced (53.1% male, 46.9% female). GPs estimated the amount of their patients with a migrant background to be 16.7% on average. About 14.7% of GPs stated having a migrant background themselves. Characteristics of the study population are summarised in Table 1.

### GPs’ problems in diagnosing dementia

**Fig. 2:**
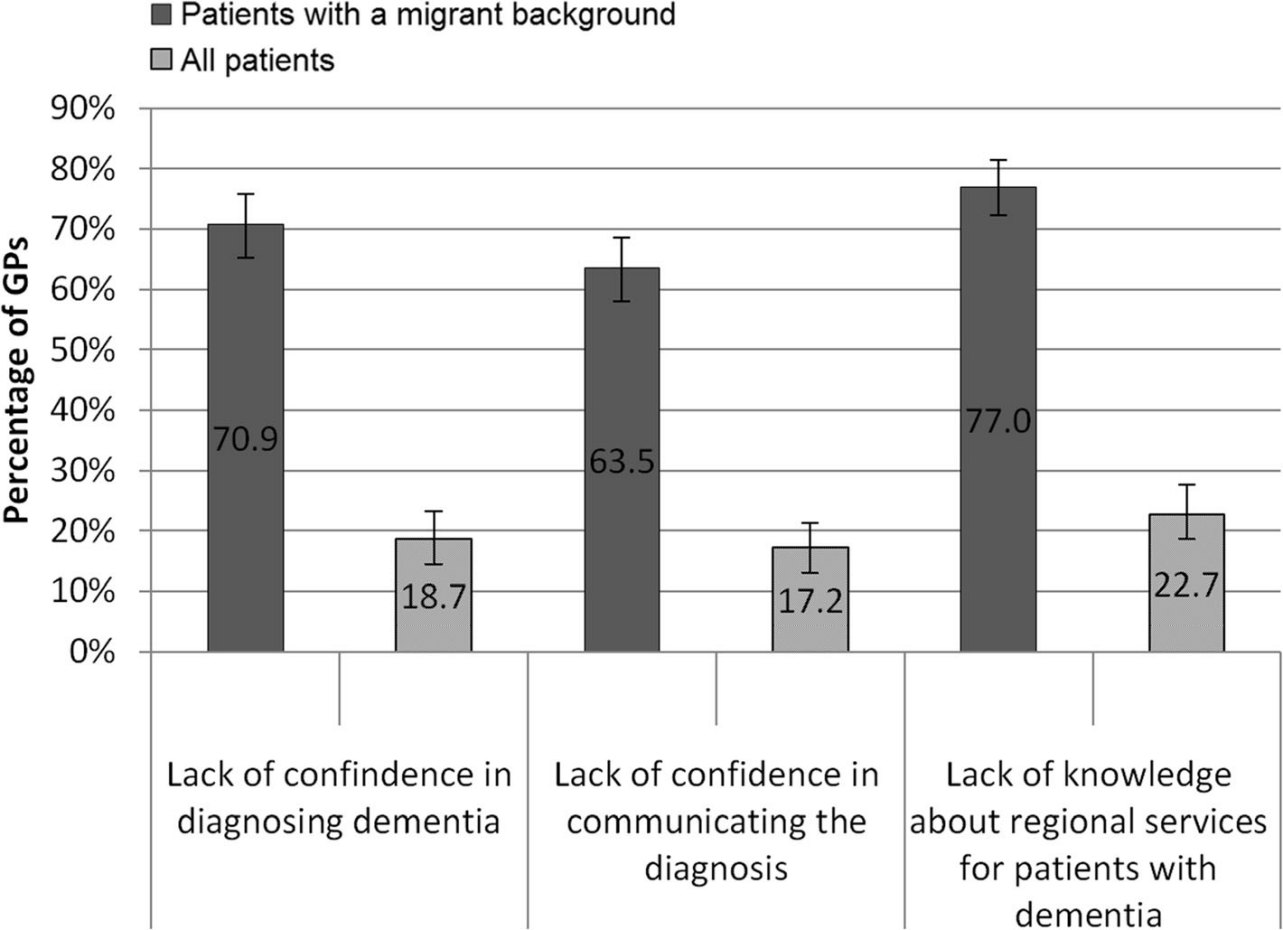
GPs’ problems in diagnostics in patients with a migrant background compared to all patients (*n* = 326)* percentage with 95%-CI, answer options “I agree” and “I rather agree”.

Ninety-six percent of GPs (*n* = 326) experienced at least one barrier in the course of diagnosing dementia in their patients with a migrant background. Because of these barriers, 88.2% reported that they were not able to treat a patient with migrant background as they wished (18.1% of them answering “often” or “very often”). Feeling “not or rather not” confident in diagnosing dementia in people with a migration background was stated by 70.9%. Only 6.7% felt very confident (Fig. 2). The comparison of this value with the confidence in diagnostics among GPs’ patients overall shows lower values of 18.7% (not confident/rather not confident). GPs aged 50 years or older, without a migrant background themselves, and treating many patients with a migrant background reported being less confident in diagnosing dementia in patients with a migrant background (Table 1). Especially female GPs with more than 20% of patients with a migrant background reported uncertainties in diagnostics. However, these associations were not found to be significant in logistic regression analysis (Table 2). Moreover 69.9% experienced being unable to perform cognitive short tests because of communication problems with patients with a migrant background. Other values comparing GPs answers are shown in Fig. 2.

### Most common barriers and information needs

**Fig. 3:**
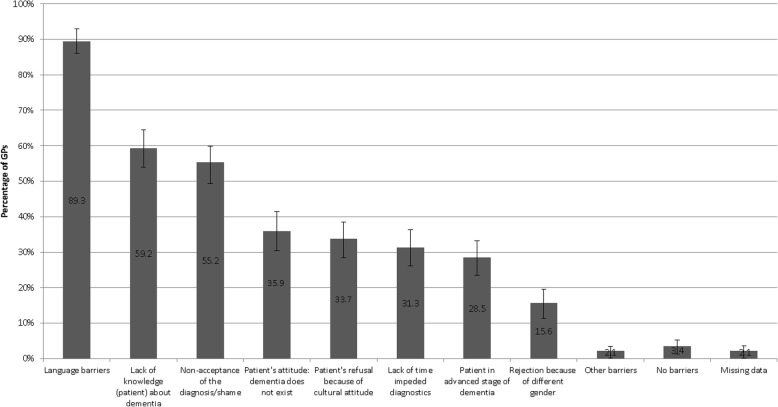
Challenges for GPs in diagnosing dementia in patients with a migrant background (n = 326)* percentage with 95%-CI

The most common barriers experienced at least once by GPs in diagnosing dementia in their patients with a migrant background are presented in Fig. 3: The most commonly reported problem was a language barrier that impeded the diagnostic process or made it impossible (89.3% of GPs). To deal with these communication problems, 90.5% of participants reported involving family members or friends of the patient as interpreters or practice staff (27.6%). 26.1% referred patients to a physician with necessary language knowledge. A share of 8.3% used the help of a professional interpreter. 8.0% provided information material in a foreign language and 7.1% referred their patient to a foreign-language service point (multiple answers possible).

70.6% of GPs expressed a demand for more information on how to better treat patients with dementia and a migrant background. Specific diagnostic tools (39.9%), dealing with language barriers (45.7%) and cultural challenges as well specialised services for patients with a migrant background (53.7%) represented key interests.

## Discussion

### Key findings and interpretation

The present study revealed a wide range of unmet challenges that GPs face in diagnosing dementia in patients with migrant background. According to GPs, these problems lead to a lack of confidence in the diagnostic process and in communicating the diagnosis. Descriptive analysis found GPs aged 50 years or older, those without a migrant background themselves and those treating many patients with a migrant background in their practices to report uncertainties more frequently. However, no proof of significance of these differences was established in logistic regression analysis. Factors impeding diagnostics are multifaceted and include language barriers between GPs and patients with a migrant background but also lack of knowledge about the syndrome and possible non-acceptance in migrants. As a consequence, nearly 90% of GPs experienced personal limitations when treating patients with a migrant background. Over 70% of GPs expressed a demand for more information on the topic. In line with international studies, our results clearly emphasise the need to support GPs in providing healthcare to patients with a migrant background.

Our study is the first in Germany to examine problems in diagnosing dementia in people with a migrant background in primary care. Results may be internationally transferable and may be an explanation for the potential underdiagnosis and late diagnosis of dementia in people with a migrant background described in former studies [3, 29, 30]. Uncertainties in diagnosing dementia and GPs’ lack of knowledge about regional services have also been reported in other studies: Cahill et al. [31] found that 30% of Irish GPs showed lack of confidence and Pathak et al. reported that more than 46% of GPs were not or not at all confident in the process of diagnosing dementia in all patients[32]. Pathak et al. report that more than half of the 380 GPs in their study were unaware of any dementia care services in their local area [32]. Turner et al. also found that more than half of GPs in their study reported lack of knowledge about dementia patients’ support groups in their area [33]. The results of the present study found an even higher proportion of GPs being uncertain in diagnosing dementia concerning patients with a migrant background. This finding suggests that these patients in particular require special attention. Our results highlight the need to prepare GPs for challenges linked to dealing with patients with a migrant background, to inform them about their options and ways of handling barriers. Cultural differences in dealing with the syndrome and risks of using non-professional interpreters should be highlighted. Ways to find information as well as regional, native-language services for patients with a migrant background should be clarified. The percentage of patients with a migrant background estimated by GPs is lower than the official statistics for the region. This result suggests that GP services are either used less frequently by patients with a migrant background in general or the migrant background of patients often goes unnoticed by GPs. There are international results that patients with a migrant background generally use healthcare services less often [6, 11, 34] and later after the onset of dementia symptoms [3, 8].

The lack of knowledge, acceptance and shame regarding dementia which physicians perceived in migrant patients is likely multicausal: the average level of education of people with a migrant background in Germany is lower than that of people without a migrant background. According to the Federal Office for Migration and Refugees (BAMF) and the German Institute for International Educational Research, the number of individuals with a lower secondary education is three times higher among foreign nationals living in Germany than in German nationals. Only 23.7% (compared to 44.2% of Germans) graduate with an A level diploma, the highest school degree in Germany [35, 36]. Since a high level of education is needed to study medicine in Germany, the on average lower educational level may be a reason for the relatively low proportion of healthcare providers with a migrant background in our study. However, there are also diverse cultural circumstances that must be taken into account: dementia and its connected diseases are often not accepted as medical problems or do not exist in some cultures [5–9]. “Forgetfulness” can be regarded as a normal consequence of aging and individuals may attempt to conceal it from others. The syndrome can be accompanied by the refusal of care, since this is regarded as a family duty [4, 8–10]. However, the role of the family in providing care can also be considered a resource as long as the family is able to deal with the situation. Feelings of shame associated with dementia and tabooing of the syndrome and other mental health impairments have already been identified in other studies [37, 38]. At this point, however, it should also be considered that cultural differences and other barriers mentioned by GPs reflect the subjective view of the GPs. Factors impeding the diagnosis of dementia such as shame and refusal may also be due to other factors such as a low level of education and poor health literacy. These barriers may be tackled by increasing knowledge about the disease [39]. In line with previous studies, these findings highlight a strong need for clear, accessible and understandable information for patients about dementia and underlying diseases [2, 10]. Providing material in different languages and native-language regional information centres and care facilities are necessary to ensure high-quality health care for the entire population. GPs could refer their patients to the centres for more information which may, in turn, reduce GPs’ workload. Currently, these multilingual service centres for dementia patients are rare in Germany.

The frequently reported language barriers that impair the diagnosis of dementia in the present study are in accordance with Australian [2] Belgian [3], Swedish [4] and European [1] studies. Dementia screening instruments like cognitive short tests are primarily language-based and not suitable for all patient groups of other cultures and native languages [1, 6, 40]. Action should be taken to develop language- and culture-independent diagnostic tools and to facilitate access to professional interpreters. In our study mainly non-professionals were reported to act as interpreters during the diagnosis of dementia, more frequently than in other medical settings that have been studied thus far [1, 41]. Since mental symptoms or disorders are often tabooed or associated with shame [5–9], non-professional interpreters such as family members or friends may distort the translation and skip the unpleasant or burdensome diagnosis or symptoms [42]. The small number of GPs who worked with a professional interpreter may be due to the lack of reimbursement in general practitioners’ practices [43]. It would be desirable to establish a pool of interpreters and to facilitate access in GP practices.

### Limitations

Although our study addressed GPs who usually come into frequent contact with migrant patients [26], some study limitations must be taken into account: Other health professionals such as neurologists and nursing staff, were not included. A response bias cannot be excluded as the responding GPs may be more interested in the topic than non-respondents. Results might differ from other parts of Germany, for example areas with a lower proportion of people with a migrant background, differing patient populations and service infrastructure. However, since characteristics of GPs, such as the age patterns, are similar to sociodemographic characteristics of GPs at national level, the results may allow generalisation [44]. Barriers and problems identified in this study may not be transferable to all migrant populations because of heterogeneous cultures, religions and views existing even within countries.

## Conclusion

Taking into account the increasing proportion of elderly people and individuals with a migrant background in the population, the development of public health measures and diagnostic tools suitable for all population groups to support GPs in their interaction with these patients is needed. The development of regional service points for dementia patients and strategies to disseminate information are desirable. Efforts to facilitate access to interpreting services and to support high quality healthcare for migrants are needed.

## Abbreviations

aOR: Adjusted odds ratio
CI: Confidence interval
Destatis: German Federal Statistical Office
DRKS: German Clinical Trials Register
GP: General practitioner
OR: Odds ratio
Ref: Reference category
SD: Standard deviation
